# The Treatment of Peri-Implant Diseases: A New Approach Using HYBENX^®^ as a Decontaminant for Implant Surface and Oral Tissues

**DOI:** 10.3390/antibiotics10050512

**Published:** 2021-04-30

**Authors:** Michele Antonio Lopez, Pier Carmine Passarelli, Emmanuele Godino, Nicolò Lombardo, Francesca Romana Altamura, Alessandro Speranza, Andrea Lopez, Piero Papi, Giorgio Pompa, Antonio D’Addona

**Affiliations:** 1 Unit of Otolaryngology, University Campus Bio-Medico, 00128 Rome, Italy; micheleantonio.lopez@gmail.com; 2 Division of Oral Surgery and Implantology, Institute of Clinical Dentistry, Department of Head and Neck, Catholic University of the Sacred Heart, Gemelli University Polyclinic Foundation, 00168 Rome, Italy; piercarmine.passarelli@unicatt.it (P.C.P.); emmanuelegodino20@hotmail.it (E.G.); nicolo.lombardo9627@gmail.com (N.L.); alesperanza121@gmail.com (A.S.); antonio.daddona@policlinicogemelli.it (A.D.); 3 Department of Oral and Maxillo Facial Sciences, Policlinico Umberto I, “Sapienza” University of Rome, 00161 Rome, Italy; francesca.altamura92@virgilio.it (F.R.A.); giorgio.pompa@uniroma1.it (G.P.); 4 Universidad Europea de Madrid, 28670 Madrid, Spain; t.v.andrea@live.it

**Keywords:** implant dentistry, peri-implantitis, mucositis, HYBENX, decontamination, oral surgery

## Abstract

Background: Peri-implantitis is a pathological condition characterized by an inflammatory process involving soft and hard tissues surrounding dental implants. The management of peri-implant disease has several protocols, among which is the chemical method HYBENX^®^. The aim of this study is to demonstrate the efficacy of HYBENX^®^ in the treatment of peri-implantitis and to compare HYBENX^®^ with other chemical agents used in the surgical treatment of peri-implantitis. Methods: The present study included a population of ten subjects with severe peri-implantitis. The procedure used in the study involves the application of HYBENX^®^ after open-flap debridement. Each patient has been followed for 12 months after a single application of the decontaminant agent. Clinical and radiographical parameters were recorded at baseline, 3 months, and 12 months after treatment completion. Results: At baseline, a mean pocket probing depth (PPD) of 7.3 ± 0.5 mm and a mean clinical attachment level (CAL) of 8.8 ± 0.8 mm was recorded. An average residual PPD of 4.2 ± 0.5 mm and a mean CAL of 5.2 ± 0.8 mm were observed after 1 year. Additionally, the average of bone gain was about 3.4 mm, with a mean marginal bone level (MBL) change from 5.8 mm (baseline) to 2.4 mm (12 months). In total, 90% of the treated implants reached the success rate after the 1-year follow-up. Only in one case out of ten treated implants was resolution of the disease not achieved. Conclusion: Clinical improvements highlight that the procedure of open-flap debridement (OFD) + HYBENX^®^ may be considered an effective technique in the treatment of peri-implantitis. From the results obtained, it can be concluded that the use of HYBENX^®^ in the surgical treatment of peri-implantitis is promising. Overall, this protocol demands further studies to better understand the role and potential benefits of HYBENX^®^ in the treatment of peri-implantitis.

## 1. Introduction

During the last few years, several branches of dentistry have undergone major developments, most notably implantology. The progress of implant therapy has resulted in a more predictable and effective implantology over the last fifty years, in both fully and partially edentulous patients [1], thus it is a viable alternative to fixed and removable partial dentures [2]. As a consequence of the increase in implant rehabilitation, a raise in peri-implant infection is expected in the future. The current available literature indicates that one of the major causes of implant failure is biofilm colonization of the implant surface [3]. There are two types of infection: the first affects only soft tissue (peri-implant mucositis) while the second results in the loss of supporting bone (peri-implantitis) [4]. Peri-implant mucositis is usually reversible and is characterized by swelling and erythema of peri-implant mucosa, associated with bleeding on probing and probing depth (PD ≥ 4 mm) without bone loss. Conversely, peri-implantitis is a pathological condition concerning all peri-implant tissues, characterized by progressive bone loss around implants. The clinical signs of peri-implantitis are deep probing depth (≥6 mm), bleeding on probing (BOP^+^), radiographic evidence of bone loss and/or suppuration [5]. The accumulation of bacteria in the soft tissue determines a chronic inflammation, which leads to the formation of a pocket of space around the implant surface and the bone and causes bone resorption. Peri-implant mucositis is deemed to be the precursor of peri-implantitis, as gingivitis is the precursor of periodontitis [6]. The early detection and elimination of risk factors are essential to avoid further progression of the disease and to ensure the maintenance of the implants over time. The 11th European Workshop on Periodontology provided new prevalence data for both diseases, estimating 43% for peri-implant mucositis and 22% for peri-implantitis [7]. Peri-implantitis is a ubiquitous disease and is directly influenced by the passage of time; the more time passes, the more implants will be affected by peri-implantitis [8]. In regard to treatment, eliminating infection and inflammation around implants is essential to resolve peri-implant disease. Peri-implant mucositis can be resolved by mechanical treatment along with increased attention to personal oral hygiene by the patient. Mechanical debridement is considered sufficient to decontaminate implants; however, it must always be accompanied by both mechanical and chemical home oral hygiene procedures. The aim of this procedure is to eliminate supra- and subgingival biofilm using curettes and ultrasonic devices. It is recommended to perform mechanical debridement through ultrasonic devices with plastic-coated tips or carbon-fiber tips. The amount of remaining plaque and calculus should be removed using titanium, carbon-fiber or plastic curettes. Finally, the implant surface should be polished with a rubber cup and non-abrasive polishing paste [9]. Chlorexidine can be used as an adjunctive therapy to mechanical debridement to prevent biofilm formation [9,10]. Nevertheless, no predictable benefits were found in the reduction of pocket depth and plaque index when chlorhexidine therapy was used in addition to mechanical debridement only [11]. The main purpose of peri-implantitis therapy is to stop the progression of the disease by acting on the causes. The treatments proposed for peri-implantitis are focused on implant surface detoxification; these strategies are based on the evidence resulting from the treatment of periodontitis [12]. A Cochrane systematic review (Esposito et al., 2012) has listed several treatment protocols for the management of peri-implantitis, however, it is unclear which protocol among the ones mentioned is the most effective [13]. The management of peri-implantitis comprises conservative (non-surgical) and surgical techniques. Many approaches have been suggested to detoxify implant surfaces, including mechanical methods, chemicals, laser and photodynamic therapies. These same mechanical procedures are also used for the treatment of peri-implant mucositis, however, in peri-implantitis therapy, their use should be subgingival to decontaminate the implant surfaces. Figuero et al. analyzed the efficacy of several nonsurgical protocols used in peri-implantitis therapy. In particular, mechanical debridement using curettes, ultrasonic devices, air-abrasive devices or lasers, alone or combined with adjunctive chemical procedures (local antibiotics, antiseptics such as chlorhexidine), have been included in the analysis. No significant benefits were found in the reduction of probing depth, whereas the positive effects on BOP were remarkable [9]. In conclusion, current evidence suggests that peri-implantitis does not respond to traditional non-surgical therapy [14]. In the case of advanced peri-implant lesion (PD > 5 mm and bone loss), the use of surgical therapy is recommended. It combines non-surgical procedures with resection and/or regenerative techniques [15]. The aim of surgical therapy in peri-implantitis is to gain access to contaminated implant surfaces in order to improve implant surface exposure and the efficacy of decontamination techniques. As a result, the greater removal of granulation tissue and pathogens is achieved to obtain reosseointegration [16,17]. A longitudinal trial analyzed clinical outcomes of surgical and non-surgical treatment in peri-implant sites after a three-year follow-up. Results showed that surgical therapy entailed a considerable decrease in probing depth score, whereas non-surgical therapy caused no significant improvement [18]. Several decontamination procedures during peri-implant surgery have been proposed, such as mechanical, chemical and laser treatments. The chemical agents proposed for decontamination procedures include citric acid, chlorhexidine gluconate, hydrogen peroxide, phosphoric acid and tetracycline. Among the various chemical methods, citric acid (CA) is one of the main agents capable of decontaminating implant surfaces. However, further studies are needed to identify the most effective concentration of the acid and the time of application during treatment. Chlorhexidine gluconate is the most widely used oral decontaminant and is also used in the decontamination of implant surfaces. Nonetheless, a comprehensive review reported that there is not enough evidence to demonstrate the superiority of chlorhexidine over other decontamination systems [19]. Hydrogen peroxide can also be used for this purpose. Tetracycline is a bacteriostatic antibiotic that inhibits protein synthesis. Case reports in humans have shown that 50 mg/mL of Tetracycline applied for 5 min after implantoplasty or air powder (AP) and followed by an autogenous bone graft or xenograft and membrane resulted in the arrest of the disease and radiographic bone fill of the peri-implant defects. In general, antibiotics are widely used in implant decontamination. Current evidence suggests more clinical benefits in the local use of antibiotics with respect to the local use of chlorhexidine, especially when the treatment is repeated over time [20,21,22]. Among all of these treatment options, HYBENX^®^ has been proposed as a decontaminant gel. HYBENX^®^ is a chemical desiccant and its use allows the selective removal of microbes and tissue debris. Its composition consists of a mixture of acidified phenolics (60% sulfonated phenolics, 28% sulfuric acid and 12% water), which guarantee a desiccant function. A good blending of its components is important to avoid harm to the surrounding healthy mucosa. The principal action of HYBENX^®^ is based on a desiccation shock debridement technology (DSD), which consists of using a new class of non-antibiotic cleanser to eradicate pathogens and the residual molecular matrix from infected tissue surfaces. Its selectivity allows for reduced bleeding of the treatment area, reduced pain, and reduced infectious load. The aim of this study is to demonstrate the efficacy of HYBENX^®^ to decontaminate the implant surface, not only in the case of mucositis but also in the case of peri-implantitis and to stimulate bone regeneration of the affected area. Additionally, the study compares open-flap debridement (OFD) + HYBENX^®^ with other chemical agents used in the surgical treatment of peri-implantitis.

## 2. Results 

In Table 1, the mean clinical parameters of pocket probing depth (PPD), marginal recession (MR), clinical attachment level (CAL) and +SD (standard deviation) are presented. The average of peri-implant pocket reduction was 3.1 mm; in percentage it corresponds to 42% less. In particular, at baseline the mean PPD was 7.3 ± 0.5 mm and after 12 months the mean PPD was 4.2 ± 0.5 mm. Additionally, at baseline the mean CAL was 8.8 ± 0.8 mm and after 12 months it changed to 5.2 ± 0.8 mm; a CAL gain of 3.6 mm, which is 41% less, was achieved. At baseline, the entire gum around implants was inflamed and swollen (BOP^+^ 100%); only 21% of implant sites were BOP^+^ 3 months after treatment. Finally, after 1 year, overall healthy gingival tissue around the dental implants was recorded; BOP^+^ occurred only in 6% of implant sites. Radiographically, the average of bone gain was about 3.4 mm, with a mean marginal bone level (MBL) change from 5.8 mm (baseline) to 2.4 mm (12 months). The mean percentage of bone fill after 1 year of treatment was 58.6%, with a minimum of 47.9% and a maximum of 69.2%. Radiographic bone level changes and bone fill are presented in Table 2. No bacterial suppuration was found in any of the cases treated during the 12 months follow-up. All baseline values were obtained immediately before surgery.

## 3. Discussion

The present study has shown that the use of HYBENX^®^ seems to keep under control peri-implantitis and may reduce clinical and radiographic parameters. The HYBENX’s ability to decontaminate implant surfaces comes from the desiccation shock debridement technology (DSD), a new chemical approach that causes complete bacterial biofilm desiccation. As a result, it can offer rapid and safe decontamination for any dental procedure. HYBENX^®^ is used in various branches of dentistry, but especially it can be used as a decontaminant in all kinds of periodontal and peri-implant procedures. According to Lauritano et al., HYBENX^®^ is an effective chemical device for use in the management of moderate to severe chronic periodontitis [23]. Isola et al. compared the use of HYBENX^®^ with scaling and root planing (SRP) versus SRP alone in the treatment of chronic periodontitis. The HYBENX^®^ group obtained a PPD reduction of 2.73 ± 0.65 and a CAL gain of 1.94 ± 0.33, while the SRP alone group had a PPD reduction of 1.8 ± 0.52 and a CAL gain of 0.74 ± 0.42. The authors concluded that SRP plus HYBENX^®^ resulted in a greater reduction in clinical, microbial, and inflammatory parameters compared to SRP alone [24]. According to other studies, the biofilm decontamination action using HYBENX^®^ is a promising technique that can be used in acute periodontal abscess and peri-implantitis treatments; all of this avoids the use of systemic or local antibiotics [25,26]. Other protocols using chemical agents for implant decontamination in the surgical treatment of peri-implantitis are described in the literature. First, studies using acids at low pH (<2) have demonstrated antimicrobial effects. Among all the acids, results on decontamination with phosphoric acid might be promising. Strooker et al. compared the implant decontamination abilities of mechanical supportive therapy with the use of phosphoric acid 35%. Although the study proved a reduction in bacterial load, no significant clinical differences compared to conventional mechanical supportive therapy were shown [27]. A randomized controlled trial compares the use of phosphoric acid 35% plus open-flap debridement (test group) versus the etching gel plus sterile saline (control group). The three-month follow-up after surgery showed that 75% of the implants in the control group and 63.3% of the implants in the test group had a disease resolution (PPD ≤ 4 mm without bleeding and/or suppuration on probing). However, no significant differences in clinical and microbiological outcomes between both groups were found; as a matter of fact, phosphoric acid offers no clear benefit in clinical outcomes [28]. Antibiotics may also be used as a surface decontaminant in peri-implantitis treatment, especially Tetracycline, which is a bacteriostatic antibiotic inhibiting protein synthesis. As a result of its antimicrobial effect it is often used in surgical peri-implantitis treatment. A 6-month randomize controlled trial compared the effects of local minocycline combined with surgical peri-implantitis treatment (test group) versus the use of a placebo and surgical treatment (control group). Clinical parameters were recorded at 1, 3 and 6 months after treatment while SBL (supportive bone level) was measured on the CBCT exam at baseline and 6 months after treatment. Overall, there was a significant difference in the changes of mean PPD between the test and control groups (2.68 ± 1.73 and 1.55 ± 1.86 mm). Conversely, no significant difference in the reduction of mean BOP between the groups was detected. One month after surgical treatment, the mean BOP in both groups was reduced to about 50%. The SBL increased at the 6-month evaluation from 5.16 ± 1.74 to 5.47 ± 1.51 mm in the control group and 6.33 ± 1.91 to 7.05 ± 1.85 mm in the test group. In the test group, treatment success (PPD < 5 mm, no bleeding/suppuration on probing, no further bone loss) was achieved in 66.7% of implants. In the control group, only 36.3% of implants showed disease resolution [29]. Chemical agents such as chlorhexidine and citric acid were not taken into account because of their cytotoxic effect on osteoblast and fibroblast when their application is not focused only on implant surface but spread to peri-implant tissues. The benefits of using HYBENX^®^ were presented by Lopez et al., who have shown that the use of this chemical agent significantly reduces the need for invasive surgery, bacterial load, patient discomfort, healing time, and the need for antibiotics, yielding a clear clinical improvement in both mucositis and severe peri-implantitis [30]. Overall, the present study proved that HYBENX^®^ is a chemical agent capable of giving excellent results in controlling peri-implantitis and reducing clinical and radiographic parameters. The use of HYBENX + OFD facilitated the implant decontamination process, resulting in a significant reduction in PPD and a bone gain in the treated sites. In total, 90% of implants led to treatment success after 1-year follow-up (PPD < 5 mm, no bleeding/suppuration on probing, no further bone loss). Only in one case out of ten implants treated was resolution of the disease not achieved. From the results obtained, it can be concluded that the use of HYBENX^®^ in the surgical treatment of peri-implantitis is promising.

## 4. Materials and Methods

The present study was conducted in accordance with the requirements of the Helsinki Declaration of 1975 as revised in 2008. Informed consent was obtained from all subjects involved in the study. The present study included a population of 10 subjects (6 females and 4 males), aged from 40 to 75 years, with severe peri-implantitis, chronically inflamed tissue around the fixture, and bleeding on probing. All the patients were followed in a time range from 2019 to 2020. Ten patients treated with HYBENX^®^ have been analyzed for this clinical study. Each patient was followed for 12 months after one single application of the decontaminant agent. The following clinical parameters were recorded using a Teflon probe at baseline, 3 months and 12 months after treatment completion: pocket probing depth (PPD), measured from the mucosal margin to the bottom of the probable pocket and evaluated at six sites around each implant (buccal, palatal/lingual, mesial, distal, mid); marginal recession (MR), measured from the implant neck to the mucosal margin and evaluated at six sites around each implant (buccal, palatal/lingual, mesial, distal, mid); clinical attachment level (CAL), probing depth plus the distance from the gingival margin to the implant neck; bleeding on probing (BOP), bleeding induced by gentle manipulation of a probe to the tissue at the depth of the gingival sulcus, recorded at six sites around implant around each implant. Additionally, digitized periapical X-rays were performed using a photostimulable phosphor system at baseline, 3 and 12 months after treatment completion in order to assess bone changes and the percentage of bone fill. A customized sensor holder (Dentsply Rinn XCP-DS^®^, York, PA, USA) was constructed to obtain a parallel X-ray projection and to achieve greater dimensional accuracy of images. In each radiograph, the following parameters were recorded at the mesial and distal aspects of the implant using the software ImageJ 1.8.0: marginal bone level (MBL), distance (in mm) between the implant shoulder and the bottom of the defect; radiographic bone fill (BF), described by Jepsen et al. [31]:

BF = (BLbaseline − BL12months)/(*IDbaseline) × 100

* Intrabony defect (ID), distance (in mm) between the bottom of the defect and the line connecting the distal and mesial interproximal bone crest. All the measurements were conducted by an external blind operator.

### 4.1. Surgical Procedure for Open Flap Debridement

1. Anesthesia. Subperiosteal anesthesia through slow infiltration using articaine (4%) with epinephrine (1:100,000);

2. Incision. Soft-tissue incisions must provide adequate access to the area of interest;

3. Debriding. Debride the exposed implant using diamond burs to smooth and polish the surface. A Teflon curette may be used to further polish and finish the surface;

4. Application of HYBENX^®^. Washing with 10 vol. hydrogen peroxide (3%) and rinsing with physiological solution, then applying HYBENX^®^ for 30 s and then rinsing properly with a physiological solution;

5. Suturing.

### 4.2. Clinical Case

**Figure 1:**
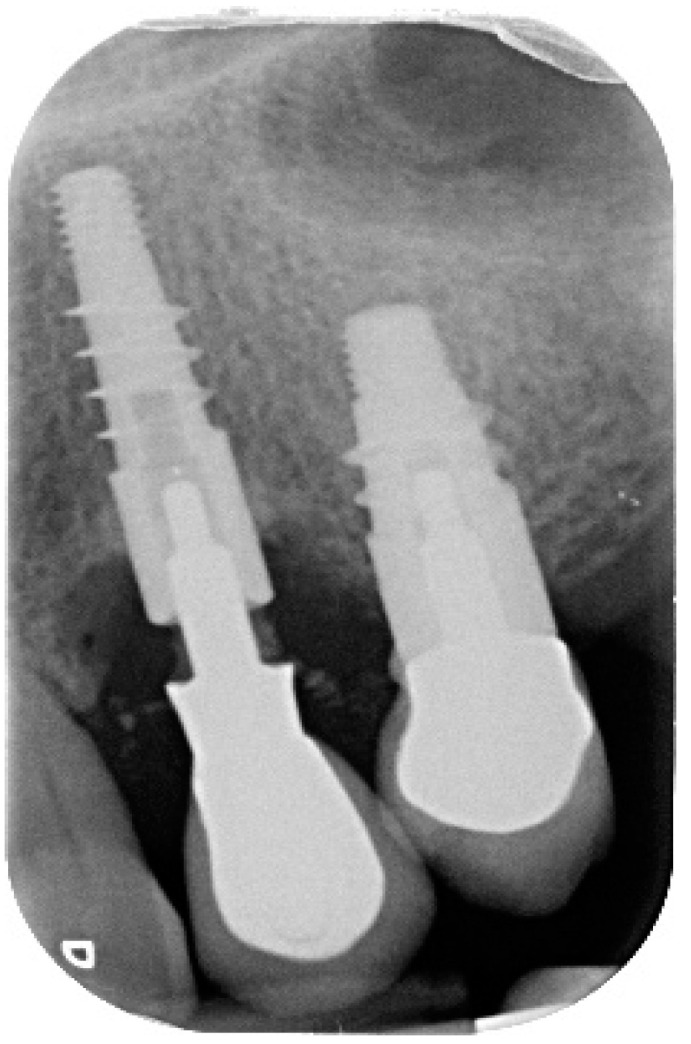
Pre-operative endoral X-ray.

**Figure 2:**
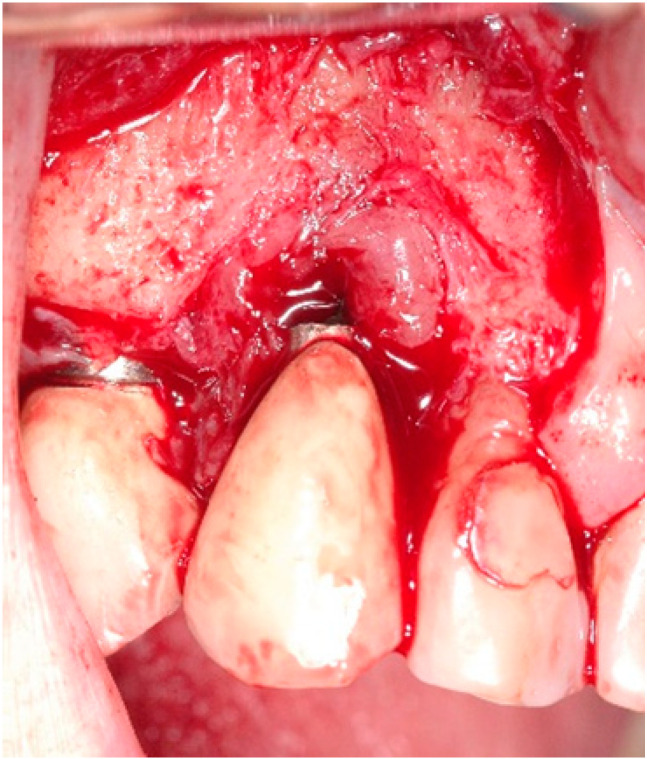
Clinical image showing inflamed tissues around the implant.

**Figure 3:**
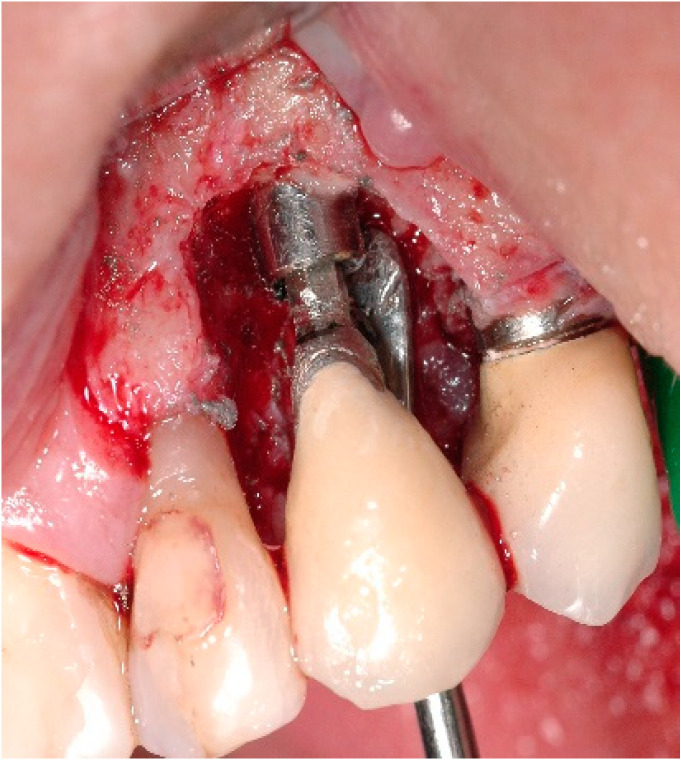
Clinical image after debriding, showing significant bone loss before treatment.

**Figure 4:**
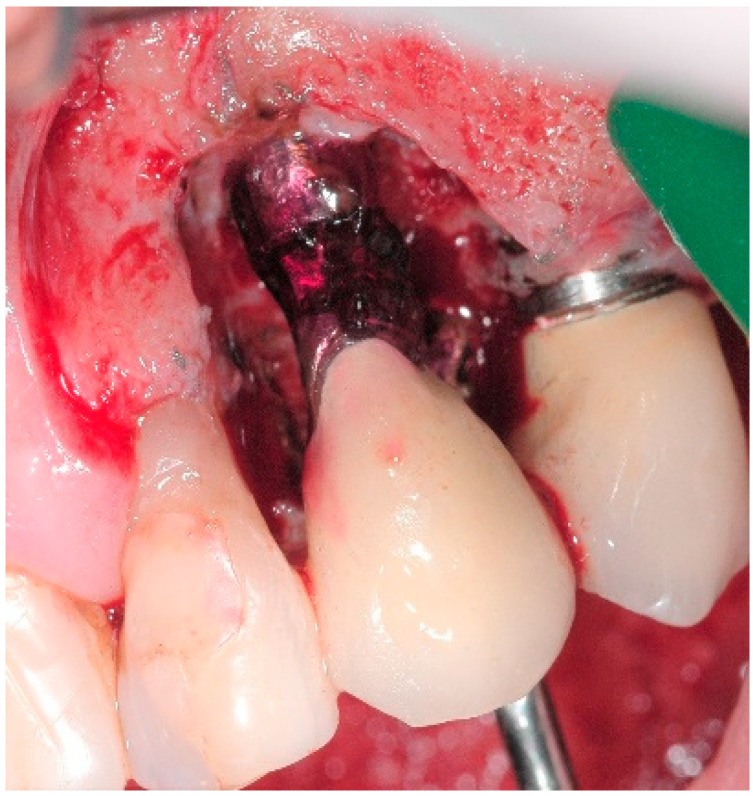
HYBENX^®^ treatment.

**Figure 5:**
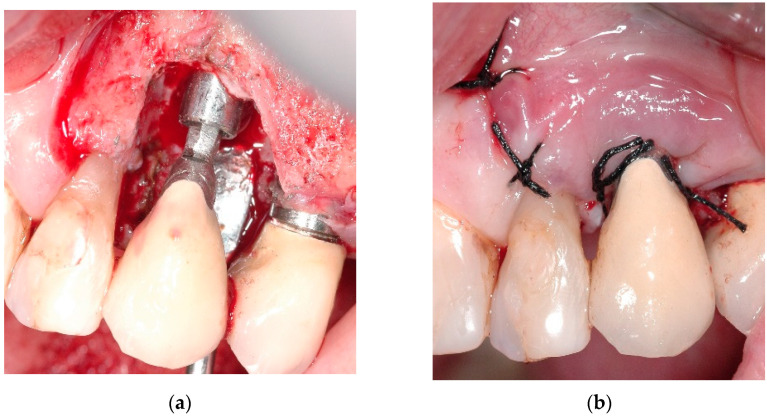
(**a**) Clinical image immediately after decontamination. (**b**) Repositioning of the mucoperiosteal flap.

**Figure 6:**
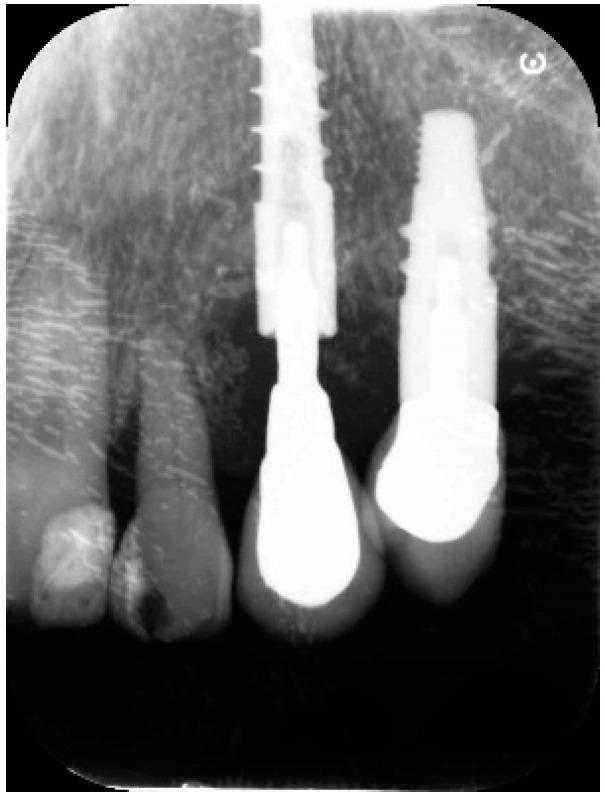
Endoral X-ray taken 3 months after treatment.

**Figure 7:**
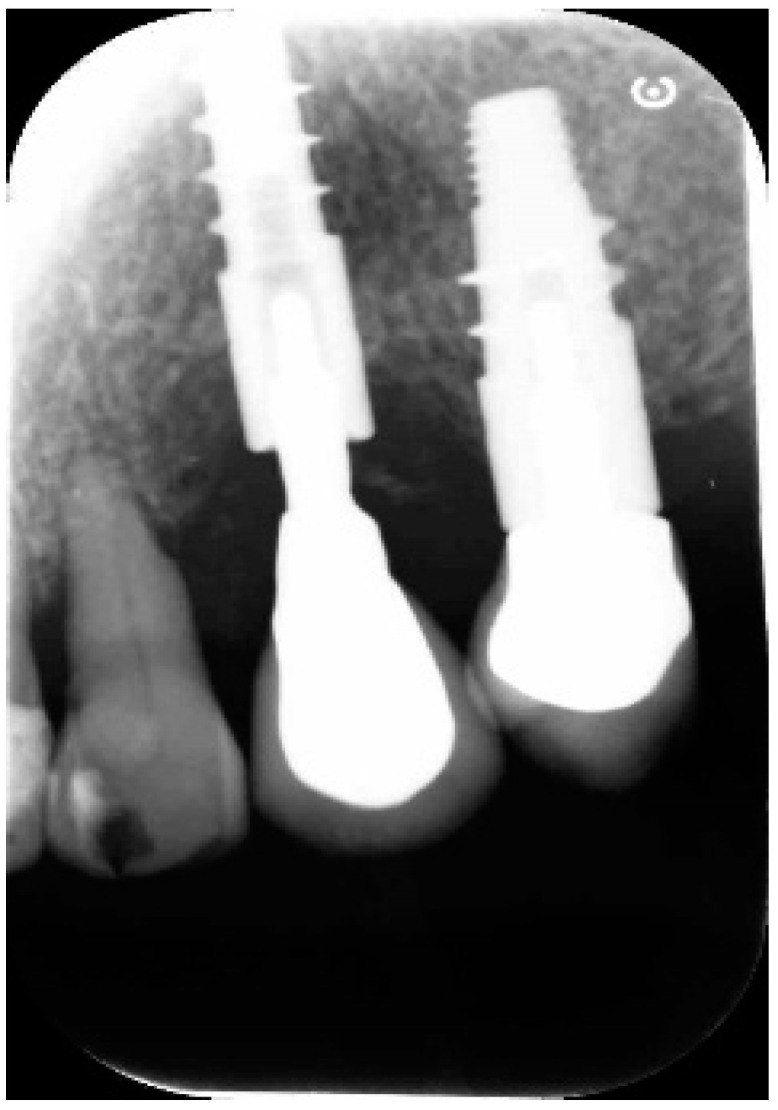
Endoral X-ray taken 12 months after treatment.

A 68-year-old female patient presented with a severe case of peri-implantitis of the fixture in position 23 (Figure 1). The patient is a non-smoker and has a good health condition. In this case it was necessary to approach the lesion with open-flap debridement. A relief incision was made in zone 22 and another in zone 24 in order to open a flap. The implant was surrounded by chronically inflamed tissue and showed a severe three-dimensional bone loss around the fixture, in particular of the vestibular alveolar bone (Figure 2). This was probably caused by problems on the implant-abutment connection since the abutment was not perfectly engaged. The first step of the procedure was to debride the exposed implant using diamond burs to smooth and polish the surface (Figure 3). In this case, a Teflon curette was also used to further polish and finish the surface. After this important step, a wash of 10 vol. hydrogen peroxide (3%) was applied, followed by a rinse with a physiological solution to remove all traces of debris and a hydrogen peroxide solution. HYBENX^®^ was applied on the implant surface for 30 s (Figure 4) and then rinsed properly with physiological solution (Figure 5). Post-operative instructions are to use a 0.2% Chlorhexidine rinse twice daily for two weeks, 800 mg of Ibuprofen for pain at the time of need, and 1gr of Amoxicillin twice daily for a week. The patient did not complain of any particular post-operative pain in the days after the treatment. After 3 months, it was not necessary to repeat the application of HYBENX^®^ because there were no signs of inflammation (Figure 6). The patient was followed for up to one year after treatment and a repeat treatment was not necessary as there was still an improvement in the condition of the mucosa and alveolar bone around the implant. Bone regeneration is also visible on the intraoral X-ray (Figure 7).

## 5. Conclusions

The ability of HYBENX^®^ to dry the surface and remove biofilm may explain the efficacy of the decontamination and subsequent clinical improvements. HYBENX^®^ treatment can be used for repeated treatments; however, in the cases reported, no further applications were required. After therapy, a marked improvement in clinical and radiographic parameters has been shown. In particular, a significant reduction in soft tissue inflammation and no bleeding on probing using a Teflon probe were noticed. Furthermore, bone gain around the fixture and a decrease in probing depth were achieved. Clinical improvements highlight that the procedure reported (OFD + HYBENX^®^) may be considered an effective technique in the treatment of peri-implantitis. Overall, this study is promising and demands further studies to better understand the role and potential benefits of HYBENX^®^ in the treatment of peri-implant disease.

## Figures and Tables

**Table 1:** Mean of clinical values: pocket probing depth, marginal recession and clinical attachment level (PPD, MR, CAL) taken at baseline and 12 months + standard deviation evaluated at six sites around each implant.

Implant Site	PPD		MR		CAL	
	Baseline	1 Year	Baseline	1 Year	Baseline	1 Year
**−11**	5.7 ± 0.5	3.0 ± 0.6	1.7 ± 0.5	0.3 ± 0.5	7.3 ± 0.8	3.3 ± 0.8
**−16**	7.7 ± 1.0	4.7 ± 0.5	1.7 ± 0.5	0.8 ± 0.1	9.3 ± 1.2	5.5 ± 0.8
**−17**	8.3 ± 0.5	5.5 ± 0.5	1.7 ± 0.5	0.7 ± 0.5	10.0 ± 0.9	6.2 ± 1.0
**−23**	6.8 ± 0.4	3.8 ± 0.5	1.2 ± 0.4	1.8 ± 0.4	8.0 ± 0.6	5.7 ± 0.5
**−25**	6.7 ± 0.5	3.9 ± 0.4	1.5 ± 0.5	1.2 ± 0.4	8.2 ± 0.4	5.0 ± 0.6
**−26**	6.7 ± 0.5	3.8 ± 0.4	1.0 ± 0.9	0.3 ± 0.5	7.7 ± 1.2	4.2 ± 0.8
**−36**	9.7 ± 0.5	4.7 ± 0.5	1.2 ± 0.4	1.7 ± 0.5	10.8 ± 0.4	6.3 ± 0.5
**−44**	7.7 ± 0.5	4.5 ± 0.5	1.3 ± 0.5	1.7 ± 0.5	9.0 ± 0.9	6.2 ± 1.0
**−46**	7.7 ± 0.5	4.7 ± 0.5	1.8 ± 0.4	1.3 ± 0.5	9.5 ± 0.5	6.0 ± 0.9
**−47**	6.5 ± 0.5	3.7 ± 0.5	1.5 ± 0.5	0.3 ± 0.5	8.0 ± 0.9	4.0 ± 0.9
**Mean Values**	6.7 ± 0.5	4.2 ± 0.5	1.5 ± 0.5	1 ± 0.4	8.8 ± 0.8	5.2 ± 0.8

**Table 2:** Marginal bone level (MBL) and bone fill (BF).

Implant Site	MBL (mm)				Bone Fill(%)	
	Baseline		1 Year		1 Year	
	Mesial	Distal	Mesial	Distal	Mesial	Distal
**−11**	4.2	3.9	1.5	1.2	64.3%	69.2%
**−16**	6.3	6.8	3.0	2.6	52.4%	61.8%
**−17**	7.1	6.2	3.7	3.1	47.9%	50.0%
**−23**	5.3	4.8	2.2	1.7	58.5%	64.6%
**−25**	5.5	5.6	2.3	1.9	58.2%	66.1%
**−26**	4.6	5.1	1.6	2.2	65.2%	56.9%
**−36**	8.3	7.8	3.3	3.5	60.2%	55.1%
**−44**	6.4	5.5	3.1	2.3	51.7%	58.2%
**−46**	6.5	6.5	3.0	3.2	53.9%	50.8%
**−47**	4.2	4.8	1.4	1.9	66.7%	60.4%
**Mean Values**	5.8	5.7	2.5	2.3	57.9%	59.3%

## Data Availability

The data presented in this study are available on request from the corresponding author.
